# Examining the associations between control (primary and secondary) appraisals and posttraumatic stress disorder symptoms in Malaysian and Australian trauma survivors

**DOI:** 10.3389/fpsyg.2023.1017566

**Published:** 2023-12-08

**Authors:** Tamsyn Reyneke, Bryan Lee, James Haoxiang Li, Shamsul Haque, Siti Zainab Abdullah, Britney Kerr Wen Tan, Belinda Liddell, Laura Jobson

**Affiliations:** ^1^Turner Institute for Brain and Mental Health and School of Psychological Sciences, Monash University, Clayton, VIC, Australia; ^2^Department of Psychology, Jeffrey Cheah School of Medicine and Health Sciences, Monash University Malaysia, Subang Jaya, Selangor, Malaysia; ^3^School of Psychology, University of New South Wales, Sydney, NSW, Australia

**Keywords:** PTSD, Malaysia, Australia, secondary control appraisals, primary control appraisals

## Abstract

**Background:**

Little research has considered the influence of culture on control appraisals in the context of posttraumatic stress disorder (PTSD).

**Objectives:**

This study aimed to investigate whether cultural group moderated the relationship between control (primary and secondary) appraisals and PTSD symptoms in trauma survivors from Western (Australian) and Asian (Malaysian) cultural contexts.

**Methods:**

Trauma survivors (107 Australian with European cultural heritage; 121 Malaysian with Malay, Indian or Chinese cultural heritage) completed an online survey assessing PTSD symptoms and appraisals of control.

**Results:**

Cultural group moderated the association between primary control and PTSD symptoms; the positive association was significant for the Australian group but not the Malaysian group. While cultural group did not moderate the association between secondary control and PTSD symptoms, there was an indirect pathway between secondary control appraisals and PTSD symptoms through interdependent self-construal for both cultural groups.

**Conclusion:**

The findings indicate that cultural group and self-construal influence the associations between different types of control appraisals and PTSD. Further research exploring the role of culture and different appraisal types in PTSD is needed.

## Introduction

Posttraumatic stress disorder (PTSD) is a disabling psychiatric condition characterized by re-experiencing symptoms, avoidance of trauma-related stimuli, negative alterations in cognition and mood, and hyperarousal (Al Jowf et al., 2022; Bryant et al., 2023). Given the debilitating nature of PTSD, considerable research has investigated factors contributing to the development, maintenance and treatment of this disorder (e.g., Tortella-Feliu et al., 2019; Forbes et al., 2020). One of the primary factors identified is maladaptive appraisals (Gómez de La Cuesta et al., 2019). While research in this area is impressive, it has been predominately conducted in Western cultures and is based on Western cultural understandings (Jobson, 2009; Bernardi et al., 2019)^.^ However, PTSD has been observed in most societies and cultures (e.g., Bahari et al., 2017; Kessler et al., 2017; Phoenix Australia, 2022; Castiglioni et al., 2023; Stein et al., 2023). Therefore, it is unclear whether the role of maladaptive appraisals in PTSD is similar across cultures and whether current understandings are applicable to trauma survivors globally. This is important to investigate to inform culturally-tailored interventions (Huey and Tilley, 2018).

Cognitive models of PTSD highlight the central role of appraisals (Ehlers and Clark, 2000). Appraisals have been of particular interest to researchers as they are identifiable and can be modified, making them ideal treatment targets (Brown et al., 2019; Gómez de La Cuesta et al., 2019). Substantial research demonstrates maladaptive appraisals play an integral role in the development and maintenance of PTSD (Brown et al., 2019; Gómez de La Cuesta et al., 2019). Appraisals of primary control (i.e., beliefs around one’s ability to prevent and control unwanted outcomes and change aspects of an event/environment (Chang et al., 1997) have received particular attention in the literature. Perceived primary control during and following trauma is associated with PTSD (Dunmore et al., 1999; Frazier et al., 2011; Padmanabhanunni and Pretorius, 2023). While this research area is well established, a significant limitation exists; researchers have predominantly focused on Western notions of control.

Culture influences how individuals appraise events (Mesquita and Walker, 2003; Jobson et al., 2022). One of the more influential theories of cultural differences, particularly regarding cognition and emotion, is the concept of self-construal (Mesquita and Walker, 2003; Markus and Kitayama, 2010; Bernardi et al., 2019). Self-construal encompasses the way in which individuals view and make meaning of themselves in relation to others (Markus and Kitayama, 2010). The independent self-construal entails viewing oneself as autonomous, self-reliant and unique from the group and an individual’s behaviors and interpretations of the world are primarily influenced by their own actions, feelings and thoughts (Markus and Kitayama, 2010). The interdependent self-construal is characterized by connectedness to and cohesion with others and an individual’s behaviors and interpretations of the world predominantly depend on their role obligations and the actions, feelings and thoughts of others in their social group (Markus and Kitayama, 2010). In Western individualistic cultures (e.g., Australian, American) the self is viewed as independent and thus such cultures emphasize a sense of primary control (Mesquita and Walker, 2003; Markus and Kitayama, 2010; Bernardi et al., 2019)^.^ In Asian collectivistic cultures (e.g., Malaysian, Chinese), the self is perceived as interdependent and as individuals are more likely to see themselves as bound to others and influenced by their environment place less value on primary control (Mesquita and Walker, 2003; Markus and Kitayama, 2010; Bernardi et al., 2019). In Western cultures, when compared to Asian cultures, primary control appraisals have been linked to stronger negative emotional reactions and anxiety (Lam and Zane, 2004; Cheng et al., 2013). Bernardi and Jobson found for European Australian trauma survivors perceived primary control was a significant predictor of PTSD symptoms with a large effect size, whereas, for Asian Australian trauma survivors this relationship was only moderate (Bernardi and Jobson, 2019). Moreover, the relationship between primary control appraisals and PTSD symptoms was determined by the extent to which an individual emphasized the independent self-construal, and the magnitude of this relationship was contingent upon one’s cultural group (Bernardi and Jobson, 2019).

Secondary control has greater value in Asian cultures (Mesquita and Walker, 2003) and proposed to have greater relevance for the mental health of those from Asian cultures (Chang et al., 1997; Mesquita and Walker, 2003; Bernardi et al., 2019). Secondary control is defined as evoking a sense of control through attempts to fit in with the environment by changing oneself (e.g., behaviors, cognitions) (Chang et al., 1997; Mesquita and Walker, 2003). Thus, being able to adapt to the situation following trauma may have greater applicability for the recovery of Asian, interdependent trauma survivors. Despite being potentially more applicable for psychological adjustment in Asian cultures, secondary control has been overlooked in PTSD research (Bernardi et al., 2019). Additionally, studies examining cultural differences in the associations between primary control and PTSD have all occurred within Western cultural contexts (Bernardi and Jobson, 2019). Thus, to date, there have been no cross-country investigations of the relationships between control appraisals and PTSD. Therefore, this study was a cross-country study focusing on trauma survivors in Australia and Malaysia. We selected Australia as an example of a Western, individualist culture valuing independent self-construal and Malaysia as representative of an Asian, collectivistic culture, to approximate Eastern, interdependent values (Timothy Church et al., 2012; Pelham et al., 2021; Schermer et al., 2023). Additionally, PTSD is common in Malaysia (Bahari et al., 2017), yet there is little research exploring whether current treatment targets (i.e., maladaptive appraisals) are applicable in this population.

**Figure 1:**
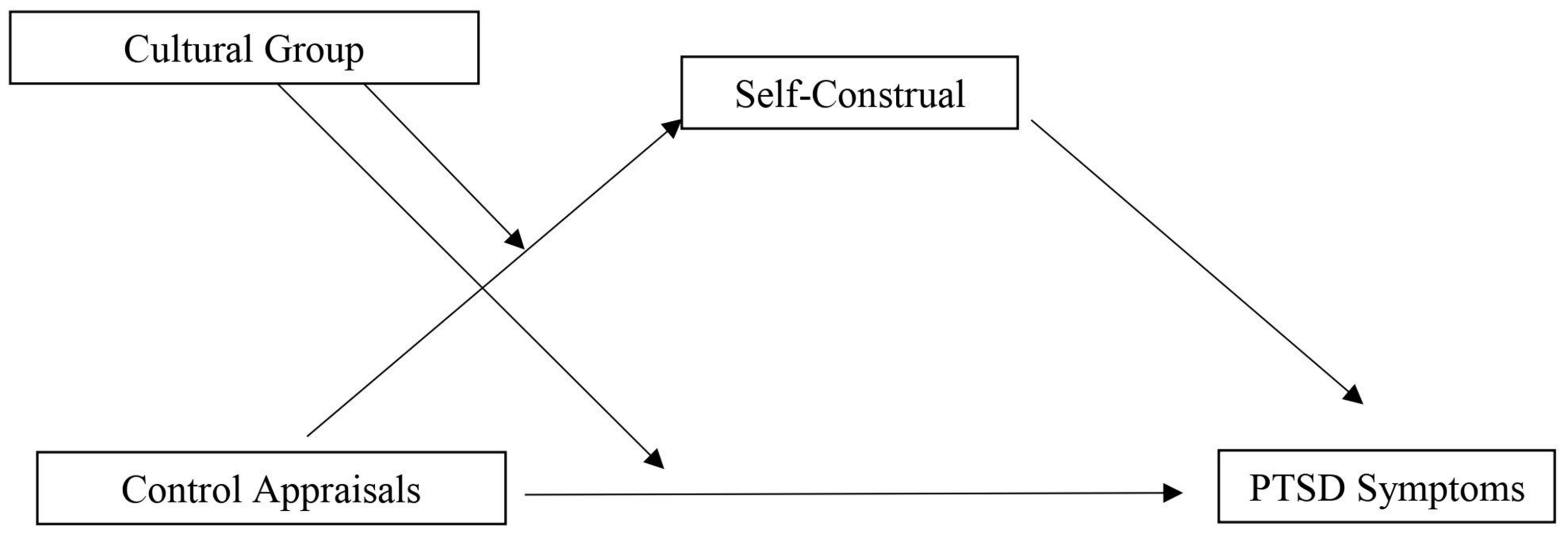
Depiction of the moderated mediation model.

This study investigated whether cultural group moderated the relationship between control (primary, secondary) appraisals and PTSD symptoms in trauma survivors from Western (Australian) and Asian (Malaysian) cultural contexts. We aimed to replicate Bernardi and Jobson (2019) using a cross-country design and aimed to extend this study by including a measure of secondary control. As in Bernardi and Jobson, we also investigated a moderating mediation model in which appraisals were the independent variable, PTSD symptoms the dependent variable, and group (i.e., Australian, Malaysian) would act as a moderator, and self-construal would function as a mediator (Figure 1). We hypothesized cultural group would moderate the relationships between control appraisals and PTSD symptoms. Specifically, we predicted the association between secondary control and PTSD symptoms would be stronger for Malaysian participants than Australian participants, while the association between primary control and PTSD symptoms would be stronger for Australian participants than Malaysian participants (Hypothesis 1). Second, we predicted that independent self-construal would mediate the relationship between primary control variables and PTSD, while interdependent self-construal would mediate the relationship between secondary control variables and PTSD (Hypothesis 2). We also explored whether cultural group moderated these mediation effects.

## Materials and method

### Design

A cross-sectional, cross-country correlational design was used. The project was co-designed by researchers in Australia and Malaysia to ensure cultural appropriateness of the study (for further details regarding study design, see Jobson et al., 2022). Monash University Human Research Ethics Committee granted approval (project number: 27577).^1^

### Participants

Of the 294 trauma-exposed responders, 66 were excluded (completed survey in <10 min: *n* = 39; scored below the conscientious response cut-off: *n* = 24; completed the survey twice: *n* = 3). The final sample included 107 Australian trauma survivors (87 female, 19 male, one did not indicate gender) and 121 Malaysian trauma survivors (92 female, 28 male, one did not indicate gender). This sample size was determined to allow analyses to be sufficiently powered. Using G*Power, based on the moderation analysis, with a moderate effect size (Bernardi and Jobson, 2019), alpha of 0.05, and 80% power, it was predetermined this study required at least 77 participants per group.

Inclusion criteria were Australian participants identifying as having European heritage (i.e., all four grandparents of European heritage); Malaysian participants identifying as having Malay, Chinese or Indian heritage (i.e., all four grandparents of Malay, Chinese or Indian heritage); being aged between 18 and 65 years; and able to complete the survey in either English or Malay. Exclusion criteria were completing the survey in under 10 min (Jobson et al., 2022); scoring below the conscientious response cut-off; completing the survey more than once; not having experienced trauma as measured by the Life Events Checklist.

### Measures

#### PTSD Checklist for the DSM-5 with Life Events Checklist

The LEC contains 17 self-report items screening for the experience of potentially traumatic events in a person’s lifetime (Weathers et al., 2013a). Participants also reported the trauma considered to be the worst event and bothers them the most (i.e., index trauma). They provide brief details about this event and the time that it occurred. The PCL-5 includes 20 self-report items measuring PTSD symptoms in response to the index trauma reported on the LEC. Responses are summed for total scores, with higher scores indicating greater PTSD symptom severity. A score ≥ 33 is indicative of probable PTSD (Weathers et al., 2013b). The PCL-5 has good psychometric properties (Weathers et al., 2013b). In this study, internal consistency was excellent (Australian: *α* = 0.95; Malaysian: *α* = 0.96).

#### Primary-Secondary Control Scale

The PSCS (Chang et al., 1997) is a 37-item questionnaire assessing appraisals of primary control (17 items; sample item “I make an effort to find ways of changing the situation”) and secondary control (20 items; sample item “I try to change myself to fit in to the situation”) in relation to an adverse life event, which was specified as the index trauma. Items are answered on 4-point Likert scales (1 = *totally disagree* to 4 = *totally agree*). Item responses are summed for each subscale. Primary control total scores range from 17 to 68 and secondary control total scores range from 20 to 80, with higher scores indicating greater degree of control beliefs. The PSCS has good psychometric properties (Chang et al., 1997). In this study, internal consistency was good for primary (Australian: *α* = 0.92; Malaysian: *α* = 0.93) and secondary control (Australian: *α* = 0.86; Malaysian: *α* = 0.88).

#### Self-Construal Scale

The SCS is a 30-item scale comprised of two sub-scales: independent and interdependent self-construal (Singelis, 1994). Items are rated on 7-point Likert scales, with scores being totaled to provide an independent and interdependent score. This scale is widely used in cross-cultural research (Singelis, 1994). In this study, internal consistency was good (Australian: independent *α* = 0.79, interdependent *α* = 0.79; Malaysian: independent *α* = 0.83, interdependent *α* = 0.77).

### Procedure

Participants were recruited using convenience sampling through online advertisements (e.g., Facebook, Gumtree). Those interested contacted the researchers and were then sent a link to the online Qualtrics survey that contained the explanatory statement. Informed consent was implied from participants’ decision to begin the survey. Participants completed the survey in English or Malay. The questionnaires were completed in the following order: PCL-5, PSCS, SCS, and demographics. Items from the Conscientious Responder Questionnaire (Marjanovic et al., 2019) were inserted randomly throughout the questionnaire to differentiate between conscientious and indiscriminate responses (those who did not score ≥ 3 correct responses were excluded from analyses; Marjanovic et al., 2019). Participants were reimbursed for their time (A$25).

### Data analysis

Data analyses were conducted using SPSS Statistics 27. No outliers or multivariate outliers were identified, and independence of residuals was assumed (Field, 2013). Several variables were not normally distributed and, as transformations did not improve normality, bootstrapping (5,000 samples) was used for all analyses (Field, 2013). Due to group differences in age, education, religion and time since trauma (see below), these variables were included as covariates in all analyses. To examine Hypothesis 1, two separate moderation regression analyses were conducted using PROCESS (model 1) (Hayes, 2017), with control appraisal (primary or secondary) as the predictor variable, PTSD symptoms the dependent variable and cultural group as the moderator. Hypothesis 2 was tested using two moderated mediation models (PROCESS model 7) (Hayes, 2017) with bias-corrected 95% confidence intervals to assess the significance of the indirect effects (self-construal) at differing levels of the moderator (cultural group). Predictor variables were control appraisals and the outcome variable was PTSD symptoms. Confidence intervals were used to determine significance of results, with confidence intervals not including 0 being considered significant.

## Results

### Group characteristics

As shown in Table 1, there were no significant group differences for PTSD or gender. There were significant group differences regarding age, time since trauma, education, and religion. The Malaysian group reported significantly greater secondary and primary control than the Australian group. 28.97% of the Australian sample (*n* = 31) and 28.93% of the Malaysian sample (*n* = 35) scored above the clinical cut-off for PTSD.

**Table 1:** Participant demographic statistics, means (standard deviations) of study variables and group differences.

	Australian	Malaysian	Group difference
Age (years)	31.66 (12.64)	25.48 (6.55)	Welch’s *t*(154) = 4.55***
Gender (female: male)^a^	87:19	92:28	χ^2^(1) = 1.00
Highest level of education^b^	26:21:38:20:2	8:10:78:19:6	χ^2^(4) = 28.50***
Religion^c^	38:56:0:6:0:7	9:19:33:38:16:6	χ^2^(5) =108.04***
Trauma type (*n*)			χ^2^(6) = 7.22
Accident/Sudden death	43	58	
Non-sexual assault	15	17	
Sexual assault	15	12	
Life-threatening illness	20	12	
War/Conflict/Kidnapping	3	2	
Natural disaster	5	12	
Other (e.g., relationship/interpersonal/family trauma)	6	8	
Time since trauma (years)	10.18 (12.38)	5.90 (5.76)	Welch’s *t* = (146) 3.27**
PTSD symptoms	22.96 (17.68)	22.68 (18.44)	*t* = (226) 0.19
Primary control	33.18 (11.37)	48.92 (11.78)	*F*(1, 205) = 56.65**, η^2^ = 0.22
Secondary control	51.66 (10.56)	63.84 (9.09)	*F*(1, 205) = 54.05**, η^2^ = 0.21

As age and time since trauma violated homogeneity of variance, Welch’s *t*-test was used. ^a^1 participant from each of the Australian and Malaysian groups did not indicate gender. ^b^Order of n shown: Secondary: Trade, technical, vocational training: Undergraduate degree: Postgraduate Degree: Other. ^c^Order of n shown: None: Christian: Buddhism and Taoism: Islam: Hinduism: Other.*p < 0.05, **p < 0.01, ***p < 0.001.

### Hypothesis 1

**Figure 2:**
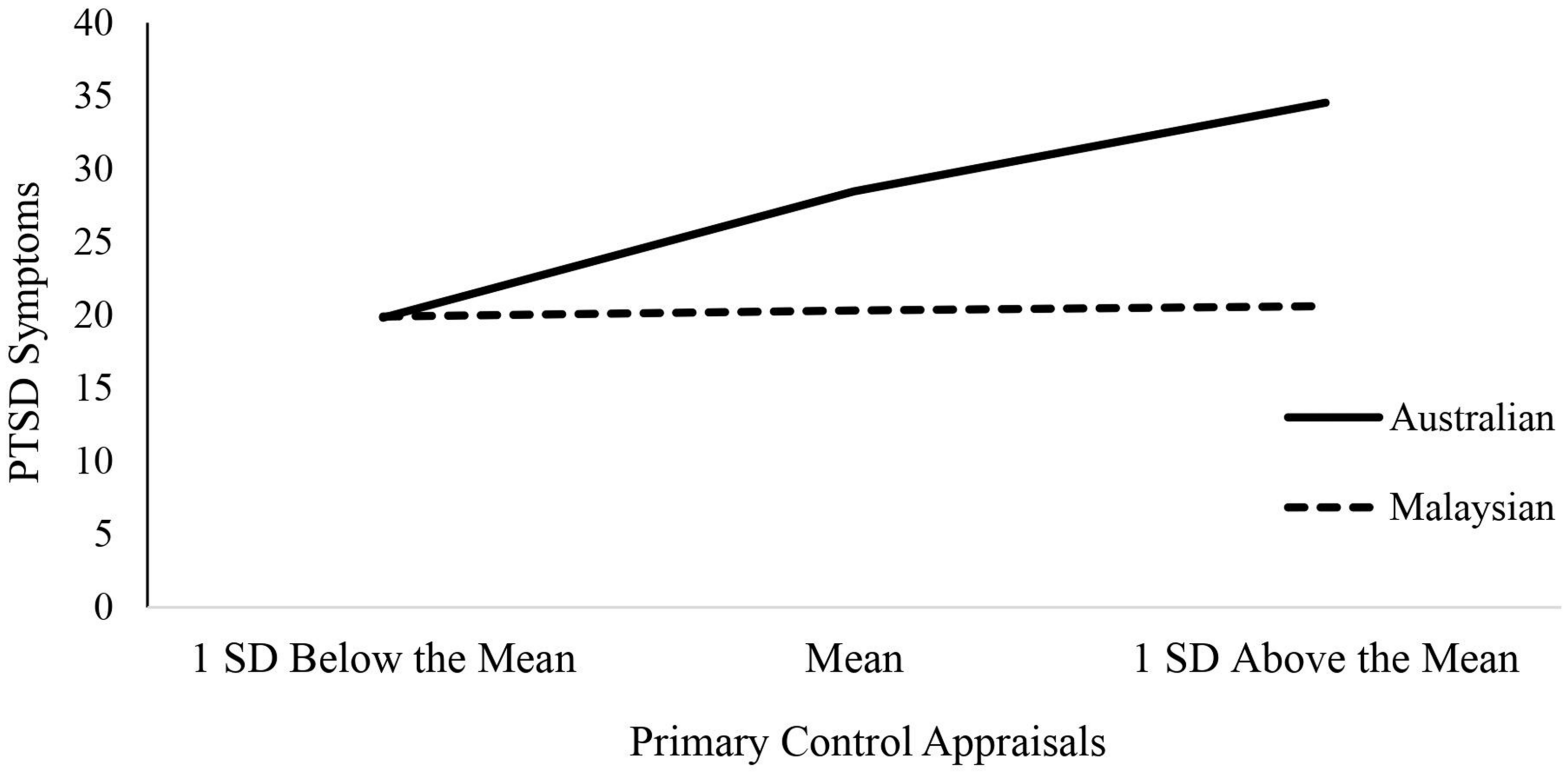
Simple slopes of posttraumatic stress disorder (PTSD) symptoms for Australian and Malaysian participants at −ISD, Mean, and +ISD of primary control.

Correlation analyses are presented in Table 2. As shown in Table 3, the moderation term was approaching significance for primary control, *R*^2^Δ = 0.02, *F*(1, 203) = 3.81, *p* = 0.05. We followed up the interaction using simple slopes analysis. As shown in Figure 2, primary control was significantly associated with PTSD symptoms for the Australian group, *B* = 0.44, *SE = 0.*16, *t* = 2.76, *p* < 0.01, 95% CI[0.12–0.74], but not the Malaysian group, *B* = 0.02, *SE = 0.*14, *t* = 0.15, *p* = 0.88, 95% CI[−0.26–0.30]. Cultural group did not moderate the association between secondary control and PTSD symptoms, *R*^2^Δ = 0.001, *F*(1, 203) = 0.31, *p* = 0.58.

**Table 2:** Bivariate spearman correlations for the Australian group (top half of the table) and the Malaysian group (bottom half of the table).

	Age	PC	SC	TST	Gender	PCL-5
Age	–	0.04	0.08	0.52***	−0.03	−0.26**
PC	0.05	–	0.49***	−0.25**	0.12	0.31**
SC	0.07	0.74***	–	−0.10	−0.09	−0.10
TST	0.10	−0.12	0.05	–	0.06	−0.35***
Gender	−0.20*	−0.17	−0.12	0.06	–	0.14
PCL-5	−0.08	0.004	−0.12	−0.07	0.12	–

PC, primary control; SC, secondary control; TST, time since trauma; PCL-5, PTSD Checklist for DSM-5. *p < 0.05, **p < 0.01, ***p < 0.001.

**Table 3:** Summary of the moderation analyses.

Variable	*B*	*SE B*	*t*	95% CI
Primary control				
Constant	6.75	13.85	0.49	−20.55, 34.06
Cultural group	9.14	9.27	0.99	−9.14, 27.41
Primary control	0.84	0.34	2.46*	0.17, 1.52
Primary control × cultural group	−0.41	0.21	1.95*	−0.83, −0.004
Secondary control				
Constant	31.35	21.93	1.43	−11.90, 75.59
Cultural group	6.76	15.20	0.44	−23.22, 36.73
Secondary control	−0.02	0.39	0.06	−0.79, 0.74
Secondary control × cultural group	−0.14	0.25	0.55	−0.64, 0.36

CI, confidence interval. *p ≤ 0.05.

### Hypothesis 2

Interdependent self-construal mediated the relationship between secondary control appraisals and PTSD symptoms for the Australian group, *B* = 0.11, *SE* = 0.05, 95% CI[0.03–0.23], and Malaysian group, *B* = 0.11, *SE* = 0.05, 95% CI[0.03–0.24]. There was no evidence of cultural group moderating this mediation, *B* < 0.01, *SE* = 0.06, 95% CI[−0.11–0.11]. Given the data was cross-sectional, we also considered the alternative model (MacCallum and Austin, 2000); interdependent self-construal would mediate the relationship between PTSD symptoms (predictor) and secondary control (outcome variable). The indirect pathway in this model was not significant. Independent self-construal did not significantly mediate the relationship between secondary control appraisals and PTSD symptoms. None of the mediation or moderating mediation analyses were significant for primary control (see Supplementary Table S1).

## Discussion

This study investigated whether cultural group moderated the relationship between control appraisals and PTSD symptoms among Australian and Malaysian trauma survivors. In support of Hypothesis 1, the moderation term for primary control was approaching significance. Follow-up analyses showed that primary control was significantly associated with PTSD symptoms for the Australian group, but not the Malaysian group. Contrary to Hypothesis 1, cultural group did not moderate the association between secondary control and PTSD symptoms. In support of Hypothesis 2, interdependent self-construal mediated the relationship between secondary control appraisals and PTSD symptoms for both cultural groups. However, contrary to Hypothesis 2, there was no evidence that independent self-construal mediated the relationship between primary control appraisals and PTSD symptoms.

Our findings, using a cross-country design, replicated Bernardi and Jobson’s findings (Bernardi and Jobson, 2019). The association between primary control and PTSD symptoms was significant for the Australian group but not the Malaysian group. This provides further support that primary control may have less relevance for posttrauma recovery among those from Asian cultures (Cheng et al., 2013; Bernardi and Jobson, 2019). Interestingly, we found a positive association between primary control and PTSD symptoms for the Australian group. This may reflect our measure assessing beliefs regarding the need to control and change the trauma experience, which taps into perceived personal responsibility for the trauma (Bernardi and Jobson, 2019). Yet, these beliefs appear less associated with PTSD symptoms for the Malaysian group. Contrary to Bernardi and Jobson, independent self-construal did not mediate the association between primary control appraisals and PTSD symptoms. Thus, there was no evidence that the relationship between primary control appraisals and PTSD symptoms was determined by the extent to which an individual emphasized independent self-construal. Further research is needed to investigate the mechanisms underpinning the cultural differences observed in the association between primary control and PTSD symptoms.

Inconsistent with our predictions, there was no evidence that secondary control appraisals were directly associated with PTSD symptoms, nor that cultural group influenced this association. There was evidence, however, that interdependent self-construal mediated the relationship between secondary control appraisals and PTSD symptoms in both cultural groups. Thus, there was some support for a role of secondary control in PTSD for those valuing interdependence. This research indicates that secondary control may play a role in posttraumatic psychological adjustment in those valuing interdependence and further research on secondary control and PTSD is warranted.

### Limitations

While this was the first cross-country study examining primary and secondary control appraisals in the context of PTSD, several limitations are worth noting. First, as the study was cross-sectional and causality cannot be inferred. This extends to our mediation analyses. While we assessed a model previously proposed in the literature (Bernardi and Jobson, 2019), it is likely that the pathways between control appraisals, self-construal and PTSD symptoms are more complex and potentially bi-directional. Thus, further research is needed. Second, the study used a community, convenience sample and the generalizability of findings to a clinical sample needs to be examined. Nevertheless, around 28% of each cultural group met the provisional diagnosis criteria for PTSD. Finally, while we considered self-construal, it is important to recognize Malaysian and Australian cultures differ in several other respects (e.g., religion, power hierarchy) that could influence findings and be considered in further research.

## Conclusion

In sum, primary control was associated with PTSD symptoms for the Australian group but not the Malaysian group and interdependent self-construal mediated the relationship between secondary control and PTSD symptoms. These findings indicate cultural group and self-construal influence the associations between different types of control appraisals and PTSD, highlighting the need for further research in this area. There are potential clinical implications. Vast research on PTSD has focused on control appraisals. However, given the Western focus of this research, the research has been on primary control. Accumulating research is now indicating primary control may have less relevance for posttrauma recovery among Asian trauma survivors. This raises questions about the appropriateness of current assessment and treatment targets that focus on appraisals, particularly appraisals of control. This study provides initial evidence for a role of secondary control in PTSD and indicates a need for further research into secondary control in the context of PTSD, alongside greater research investigating appraisal types associated with PTSD among Asian trauma survivors.

## Author contributions

LJ, SH, and BLi conceived of the presented idea. TR, BLe, JL, SA, and BT set up the study and collected the data. LJ and SH supervised the project. TR conducted the analyses and drafted the first version of the manuscript. All authors discussed the results and contributed to the final manuscript.

## Footnotes

^1^The data collected in this sample was part of a larger project that has been reported elsewhere (doi: 10.3390/ijerph19031163) (Jobson et al., 2022).
