# Supplementary material for: Examining the associations between control (primary and secondary) appraisals and posttraumatic stress disorder symptoms in Malaysian and Australian trauma survivors
Source: Front Psychol. 2023 Dec 8;14:1017566. doi: 10.3389/fpsyg.2023.1017566 (PMC10739294; doi:10.3389/fpsyg.2023.1017566)
Supplement: Supplementary file 1 [file Table_1.docx]

**Supplemental Table 1**

*Summary of the Moderating Mediation Analyses*

| Variable | *B* | *SE B* | *t* | 95% CI |
| --- | --- | --- | --- | --- |
| **Primary Control** | ­­­­­­­ |  |  |  |
| Primary Control-Independent Self Construal^a^ | -.02 | .01 | -1.14 | -.05-.01 |
| Primary Control-Interdependent Self Construal^b^ | .003 | .01 | .23 | -.02-.03 |
| Primary Control-PTSD Symptoms | .10 | .10 | 1.02 | -.10-.03 |
| Independent Self-Construal-PTSD Symptoms | -4.01 | 1.60 | -2.51* | -7.15--.86 |
| Independent Self-Construal-PTSD Symptoms | 3.35 | 1.92 | 1.75 | -.44-7.13 |
| Primary Control-Independent Self-Construal-PTSD Symptoms (Australian) | .004 | .03 | - | -.05-.07 |
| Primary Control-Independent Self-Construal-PTSD Symptoms (Malaysian) | -.06 | .04 | - | -.16-.0004 |
| Moderating Mediation Effect | -.06 | .05 | - | -.20-.01 |
| Primary Control-Interdependent Self-Construal-PTSD Symptoms (Australian) | .04 | .03 | - | -.01-.13 |
| Primary Control-Interdependent Self-Construal-PTSD Symptoms (Malaysian) | .07 | .04 | - | -.01-.16 |
| Moderating Mediation Effect | .03 | .03 | - | -.03-.10 |
| **Secondary Control** |  |  |  |  |
| Secondary Control-Independent Self Construal^c^ | -.01 | .02 | -.81 | -.05-.02 |
| Secondary Control-Interdependent Self Construal^d^ | .02 | .01 | 1.39 | -.01-.05 |
| Secondary Control-PTSD Symptoms | -.30 | .12 | -2.55* | -.54--.07 |
| Independent Self-Construal-PTSD Symptoms | -2.74 | 1.60 | -1.72 | -5.89-.41 |
| Independent Self-Construal-PTSD Symptoms | 5.38 | 1.90 | 2.83* | 1.63-9.13 |
| Secondary Control-Independent Self-Construal-PTSD Symptoms (Australian) | -.02 | .03 | - | -.08-.03 |
| Secondary Control-Independent Self-Construal-PTSD Symptoms (Malaysian) | -.07 | .05 | - | -.19-.01 |
| Moderating Mediation Effect | -.05 | .05 | - | -.19-.02 |
| Secondary Control-Interdependent Self-Construal-PTSD Symptoms (Australian) | .11 | .05 | - | .02-.23 |
| Secondary Control-Interdependent Self-Construal-PTSD Symptoms (Malaysian) | .11 | .05 | - | .02-.23 |
| Moderating Mediation Effect | .004 | .05 | - | -.11-.11 |

*Note: CI* = confidence interval. **p* < .05. *^a^* Interaction with cultural group - *R^2^*Δ=.01, *F*(1,203)=3.07, *p* = .08; *^b^* Interaction with cultural group - *R^2^*Δ=.05, *F*(1,203)=1.24, *p*=.27;

*^c^* Interaction with cultural group - *R^2^*Δ=.01, *F*(1,203)=3.39, *p*=.07;*^d^* Interaction with cultural group - *R^2^*Δ<.001, *F*(1,203)=.01, *p*=.94.
